# Performance of a single-use gastroscope for esophagogastroduodenoscopy: Prospective evaluation

**DOI:** 10.1055/a-2271-2303

**Published:** 2024-03-18

**Authors:** Koen van der Ploeg, Pieter J.F. de Jonge, Wim J. Lammers, Arjun Dave Koch, Margreet C. Vos, Vemund Paulsen, Lars Aabakken, Marco Bruno

**Affiliations:** 1 6993 Department of Gastroenterology and Hepatology, Erasmus MC, Rotterdam, Netherlands; 2 6993 Department of Medical Microbiology and Infectious Diseases, Erasmus MC, Rotterdam, Netherlands; 3 72481 Department of Gastroenterology and Hepatology, Erasmus MC Cancer Centre, Rotterdam, Netherlands; 4 155272 Department of Clinical Medicine, Oslo University Hospital, Oslo, Norway; 5 6305 Institute of Clinical Medicine, University of Oslo, Oslo, Norway; 6 155272 Department of Transplantation Medicine, Oslo University Hospital, Oslo, Norway

**Keywords:** Endoscopy Upper GI Tract, Hygiene, Quality and logistical aspects, Performance and complications

## Abstract

**Background and study aims** Reprocessing reusable endoscopes is challenging due to their non-sterilizable nature. Disinfection has been shown to have a significant risk of failure with serious consequences. Single-use endoscopes can eliminate contamination risk and reduce workflow delays caused by reprocessing. This study evaluated the clinical performance of single-use gastroscopes in patients undergoing esophagogastroduodenoscopy (EGD).

**Patients and methods** In this case series, 60 patients underwent EGD using single-use gastroscopes, with 34 procedures in the endoscopy department and 26 in the intensive care unit. The primary outcome was successful completion of the intended EGD objective. Furthermore, certified endoscopists assessed device performance on a five-point Likert scale (ranging from 1-"much worse" to 5-"much better"), considering their experience with a reusable gastroscope.

**Results** Successful completion of EGDs using only the single-use gastroscope was achieved in 58 of 60 cases (96.7%). In two cases, crossover to an ultra-slim endoscope was necessary to either reach the esophageal stenosis or to transverse the stenosis. Overall satisfaction was rated as comparable to reusable scopes in 51 of 56 cases (91.1%) and inferior in five cases (8.9%). The lower weight of the single-use gastroscope was rated as superior in 42 of 60 cases (70.0%). Drawbacks included reduced image quality (23 of 45 cases; 51.1%). Feedback included the absence of a freeze button, lens cleaning issues, and small image size.

**Conclusions** Single-use gastroscopes exhibited a high EGD completion rate and effectiveness for various indications. Further research should focus on evaluating the implementation of single-use gastroscopes in a comprehensive context, considering clinical effectiveness, costs, and environmental impact.

## Introduction

Esophagogastroduodenoscopy (EGD) is an important tool in diagnosis and treatment of upper gastrointestinal-disorders. The indications for EGD are diverse, comprising both diagnostic and interventional purposes such as dysphagia, gastroesophageal reflux disease, nasojejunal tube placement, dilatation of esophageal strictures, and treatment of upper gastrointestinal bleeding 1 . EGD is a common procedure and is performed around 6.1 million times annually in the United States 2 . Complications of EGD are rare, but include perforation, bleeding, aspiration, and infection 3 .

Endoscopy-associated infection (EAI) can be either endogenous, due to translocation of the patient’s own microbial gut flora, or exogenous as a result of contaminated equipment. The latter has received increased attention because of several outbreaks reported in past decades 4 . Endoscopes require an extensive cleaning process in which reprocessing protocol can be breached and endoscope damage or biofilm can prevent proper disinfection 5 6 . Endoscopes can only be sterilized with ethylene oxide (EtO), which has been shown to end outbreaks but also carries carcinogenic and mutagenic risks 7 . Therefore, sterilization with EtO demands specific environmental and construction prerequisites, making it time-consuming and limiting its applicability 8 . Also, in a randomized controlled trial, the addition of EtO sterilization to high-level disinfection did not improve contamination rates 9 . Many of the published outbreaks involved contaminated duodenoscopes 4 . However, outbreaks due to contaminated gastroscopes have been described, resulting in seven reported clinical infections 10 11 12 . The prevalence of contaminated patient-ready gastroscopes has been less frequently studied compared with that in duodenoscopes. A recent meta-analysis reported a contamination rate of 28.2%, based on six different studies 13 .

The precise risk of EAIs in gastroscopes, however, is unknown. It is likely that EAIs are underreported due to a lack of recognition of EAIs caused by susceptible microorganisms, limited microbiological surveillance conducted after endoscopy procedures, and the potential long duration between endoscopy and infection onset. Another explanation for failure to report recognized EAIs could be the lack of a mandatory registration system reduces the likelihood that an EAI will be reported once recognized.

In response to reported cases of EAI, the development of single-use endoscopes has gained momentum. Utilization of single-use endoscopes eliminates risk of exogenous EAIs. Their use also provides additional advantages, such as streamlining workflow by eliminating the time required for endoscope reprocessing and enabling endoscopic procedures outside regular working hours without the need for reprocessing personnel. In addition, single-use endoscopes are associated with faster and enhanced product refinement and development through successive iterations, along with the possibility of tailoring devices for specific tasks. Furthermore, single-use endoscopes and their processors are lighter and smaller, making them more portable. This streamlines endoscopic procedures in settings beyond the Endoscopy Department, including the Intensive Care Unit (ICU) and remote locations, eliminating the need for post-procedure endoscope cleaning facilities. However, it is important to consider the potential benefits of single-use endoscopes in the context of increased costs and their environmental impact 14 .

Multiple single-use duodenoscopes have already been introduced to the market, and their performance seems to be comparable to that of reusable duodenoscopes 15 16 . Ambu is the first company that has produced a single-use gastroscope (Ambu aScope Gastro). The aScope Gastro recently received European Certification (CE), but no studies of its performance for a broad range of EGD indications have been published. The aim of this study was to assess the performance of a single-use gastroscope in adult patients undergoing EGD.

## Patients and methods

### Study design

This study employed a multicenter, prospective, observational, case series design to assess performance of the CE-certified single-use gastroscope, the Ambu aScope Gastro, in accordance with its intended use. The participating centers included the Erasmus MC University Medical Center Rotterdam (Erasmus MC) and Oslo University Hospital – Rikshospitalet (OUS).

### Patient selection and informed consent

A total of 60 patients were included in this study, with 34 patients recruited from the Erasmus MC and 26 patients from the OUS. At the Erasmus MC, all eligible patients underwent EGD in the Endoscopy Department. At the OUS, only patients admitted to the ICU were considered eligible. Eligible patients were individuals aged ≥ 18 years who were scheduled for an EGD at the specified locations. Patients who were terminally ill or those who had participated in other studies that could interfere with the outcomes of this study did not qualify for participation. Patients scheduled for an EGD for Barrett's esophagus surveillance were omitted per guideline requiring a high-definition endoscope 17 . The study was approved by the Medical Research Ethics Committee of Erasmus MC (MEC-2022–0285). All Dutch patients provided informed consent. In accordance with Norwegian law, the study activities conducted at OUS were regarded as a quality assurance exercise and, therefore, informed consent was not required.

### Device description

The Ambu aScope Gastro is a sterile single-use gastroscope with a working length of 1030 mm and a 2.8-mm working channel that is compatible with commonly used endotherapy instruments. The gastroscope is used in conjunction with the Ambu aBox 2 (aBox 2) display and processing unit, which includes a touchscreen monitor. The aBox 2 features a Full HD 12.8-inch color liquid-crystal display, with a total height of 27.8 cm width of 33 cm, and weight of 8 kg.

### Setting

At Erasmus MC, all EGDs were conducted in the same room in the Endoscopy Department. Each procedure involved one endoscopist and two endoscopy nurses. The aBox 2 was linked to a video distribution device, enabling transmission of the video signal to two extra screens, typically employed during EGDs using reusable gastroscopes. Patients were positioned on their left side and given the option of sedation (midazolam and fentanyl) for the procedure. EGDs at the OUS were performed at in patient rooms in the ICU. One endoscopist, one endoscopy nurse, and one ICU nurse were present. Only the screen of the aBox 2 was used, which was positioned across the bed. Patients were mainly treated in supine position with their heads tilted toward the left side; occasionally, patients were placed on their left side. Most ICU patients were already deeply sedated; in some cases, additional sedation was given for the procedure.

### Procedure and evaluation

All EGDs were conducted in accordance with their respective indications, without any additional study interventions except for using single-use gastroscopes. The procedures were exclusively conducted by certified endoscopists, two at Erasmus MC and five at OUS. Each endoscopist had performed more than 1000 EGDs during their career and possessed expertise in advanced endoscopy techniques such as endoscopic retrograde cholangiopancreatography, endoscopic ultrasound, or endoscopic mucosal resection. None of the endoscopists had previous experience with the Ambu aScope Gastro. One endoscopist from Erasmus MC and two from OUS had experience with single-use duodenoscopes (Ambu or Boston Scientific). Furthermore, all endoscopists had prior experience with the SpyGlass (Boston Scientific), a single-use cholangioscope. After each procedure, the performing endoscopist completed a questionnaire to assess various aspects of single-use gastroscope performance compared with prior experience with a reusable gastroscope. The endoscopist made the decision if the aspect could be rated based on the EGD that had been performed. If the single-use gastroscope could not fulfill the intended purpose of the EGD, the endoscopist switched to a reusable endoscope to complete the procedure. The Erasmus MC used reusable gastroscopes from either the Olympus 180 or the Olympus 190 series, while the OUS utilized gastroscopes exclusively from the Olympus 190 series.

### Study endpoints

The primary endpoint of the study was frequency with which an endoscopist successfully achieved the intended diagnostic or therapeutic goals during an EGD. Diagnostic goals included inspecting the upper digestive tract for esophageal dysphagia, ulcer follow-up, or malignancy screening. Therapeutic goals encompassed varices treatment, nutritional tube placement, and dilation of esophageal stenosis. Secondary endpoints encompassed qualitative assessment of the single-use gastroscope in comparison with prior experiences with reusable devices, utilizing a comprehensive 5-point Likert scale (ranging from 1 – “much worse” to 5 – “much better”). This assessment covered various dimensions including image quality, handling aspects, and technical performance parameters such as suction efficiency and passage of accessories through the working channel. Diagnostics and therapeutics that were performed during the EGD were also rated. In addition, procedure duration was monitored and instances of transitioning to reusable endoscopes were recorded.

### Statistical analyses

All statistical analyses were performed using R version 4.1.3 18 . Categorical variables are presented as absolute and relative frequencies, whereas continuous variables are reported as median with first quartile and third quartile (Q1, Q3) or mean and standard deviation (SD). Due to the study design, differences in populations between the study centers, and the ordinal nature of the data, no statistical tests were conducted.

## Results

Sixty patients were included in this study, of whom 34 were from the Endoscopy Department at Erasmus MC and 26 from the ICU at OUS. Among them, 38 (63.3%) were male and the median age was 61.5 years (range 53.5–73.3). Notably, patients recruited from OUS were predominantly male (20 of 26; 76.9%) and younger (median age 57.0 years (range 42.3–68.5)) in comparison with those from Erasmus MC, where 18 of 34 (52.9%) were male and the median age was 66.0 years (range 57.5–75.0) ( Table 1 ). Furthermore, the patient cohort at Erasmus MC exhibited a higher prevalence of relevant medical histories potentially influencing the EGD procedure. Specifically, a greater proportion of these patients had a history of upper gastrointestinal surgery (4 of 34; 11.8% vs. 1 of 26; 3.8%), esophageal stenosis (8 of 34; 23.5% vs. 0 of 26; 0%), and portal hypertension (4 of 34; 11.8% vs. 2 of 26; 7.7%). History of upper gastrointestinal surgery included Roux-Y-gastrectomy (OUS, 1 of 26, 3.8%), esophagectomy with gastric conduit reconstruction (Erasmus MC 3 of 34, 8.8%) and gastric bypass (Erasmus MC, 1 of 34, 2.9%).

**Table:** **Table 1** Patient characteristics.

	Total	Erasmus MC	Oslo University Hospital
Patients, n	60	34	26
Patient's age, years, median (Q1,Q3)	61.50 (53.50,73.25)	66.00 (57.5,75.0)	57.00 (42.3,68.5)
Male gender, n (%)	38 (63.3)	18 (52.9)	20 (76.9)
Medical history, n (%)			
Upper gastrointestinal surgery	5 (8.3)	4 (11.8)	1 (3.8)
Hiatal hernia	2 (3.3)	1 (2.9)	1 (3.8)
Esophageal stenosis	8 (13.3)	8 (23.5)	0 (0)
Portal hypertension	6 (10.0)	4 (11.8)	2 (7.7)
Sedation, n (%)			
General anesthesia	18 (30.0)	0 (0.0)	18 (69.2)
Propofol	2 (3.3)	0 (0.0)	2 (7.7)
Midazolam and fentanyl	25 (41.7)	23 (67.6)	2 (7.7)
Midazolam	7 (11.7)	4 (11.8)	3 (11.5)
None	8 (13.3)	7 (20.6)	1 (3.8)
Gloucester Comfort Scale, n (%)			
Not registered	3 (5.0)	3 ( 8.8)	0 (0.0)
Mild	3 (5.0)	3 ( 8.8)	0 (0.0)
Minimal	24 (40.0)	20 (58.8)	4 (15.4)
Moderate	3 (5.0)	2 ( 5.9)	1 (3.8)
No discomfort	9 (15.0)	6 (17.6)	3 (11.5)
Not applicable (general anesthesia)	18 (30.0)	0 ( 0.0)	18 (69.2)
Q, quartile.			

## Performance

**Fig. 1:**
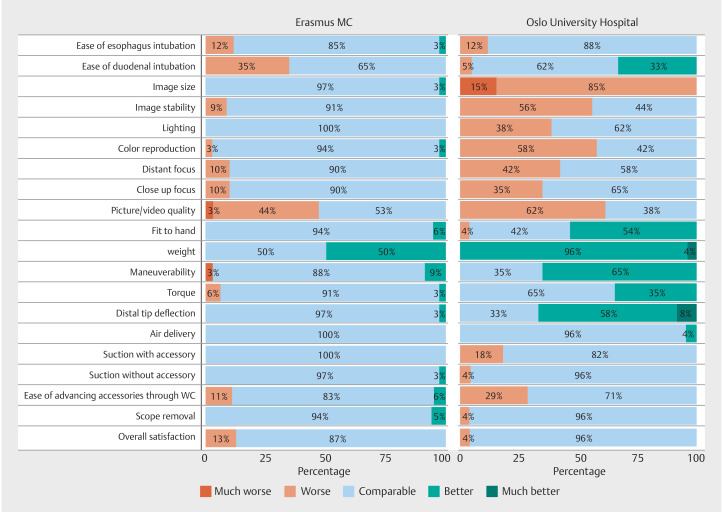
Performance rating of single-use gastroscope according to a five-point Likert scale. WC, working channel endoscope

Seven endoscopists conducted the procedures; two at the Erasmus MC and five at the OUS. In 58 of 60 cases (96.7%), the endoscopists achieved the intended diagnostic or therapeutic goals, completing the EGD without the need for a crossover. In two cases, the intended goals could not be achieved with a single-use gastroscope alone due to its diameter. Consequently, a crossover to an ultra-slim endoscope was necessary to reach or traverse the esophageal stenosis. Half of the EGD indications (30 cases) were primarily therapeutic, with a higher proportion observed at OUS (18 of 26 cases, 69.2%) compared with the Erasmus MC (12 of 34 cases, 35.3%). EGD characteristics and indications are listed in Table 2 . After every EGD, the performance of the single-use gastroscope was rated. The results of the qualitative assessment are illustrated in Fig. 1 .

**Table:** **Table 2** Esophagogastroduodenoscopy procedure characteristics and primary indication.

	Total	Erasmus MC (n = 34)	Oslo University Hospital (n = 26)
EGD location		Endoscopy Department	Intensive Care Unit
Number of endoscopists, n	7	2	5
Number of procedures per endoscopist (mean [SD])	8.6 [6.6]	17 [0]	5.2 [4.5]
Minutes per procedure, n (median [Q1,Q3])	10.00 [6.00,5.25]	7.00 [5.0,9.8]	15.50 [15.0,24.8]
Primary therapeutic indication EGD, n (%)	30 (50.0)	12 (35.3)	18 (69.2)
EGD indication, n (%)			
Nutritional tube placement	10 (16.7)	0 (0.0)	10 (38.5)
(Suspected) gastrointestinal bleeding	11 (18.3)	5 (14.7)	6 (23.1)
Treatment esophageal stricture	9 (15.0)	9 (26.5)	0 (0.0)
Varices treatment and surveillance	8 (13.3)	5 (14.7)	3 (11.5)
Follow-up (achalasia, post-surgery, ulcer)	6 (10.0)	5 (14.7)	1 (3.8)
Esophageal dysphagia	3 (5.0)	3 (8.8)	0 (0.0)
Screening for malignancy	2 (3.3)	2 (5.9)	0 (0.0)
Esophagitis assessment	2 (3.3)	1 (2.9)	1 (3.8)
Gastric conduit stenosis	2 (3.3)	2 (5.9)	0 (0.0)
Other (stent removal, necrosectomy, LAMS cleaning, duodenal biopsies)	7 (11.7)	2 (5.9)	5 (19.2)
Completed procedure with single-use gastroscope, n (%)	58 (96.7)	32 (94.1)	26 (100.0)
Failure to complete procedure intended EGD goal, n (%)	2 (3.3)	2 (5.9)	0 (0.0)
EGD, esophagogastroduodenoscopy; LAMS, lumen-apposing metal stent; Q, quartile; SD, standard deviation.			

**Fig. 2:**
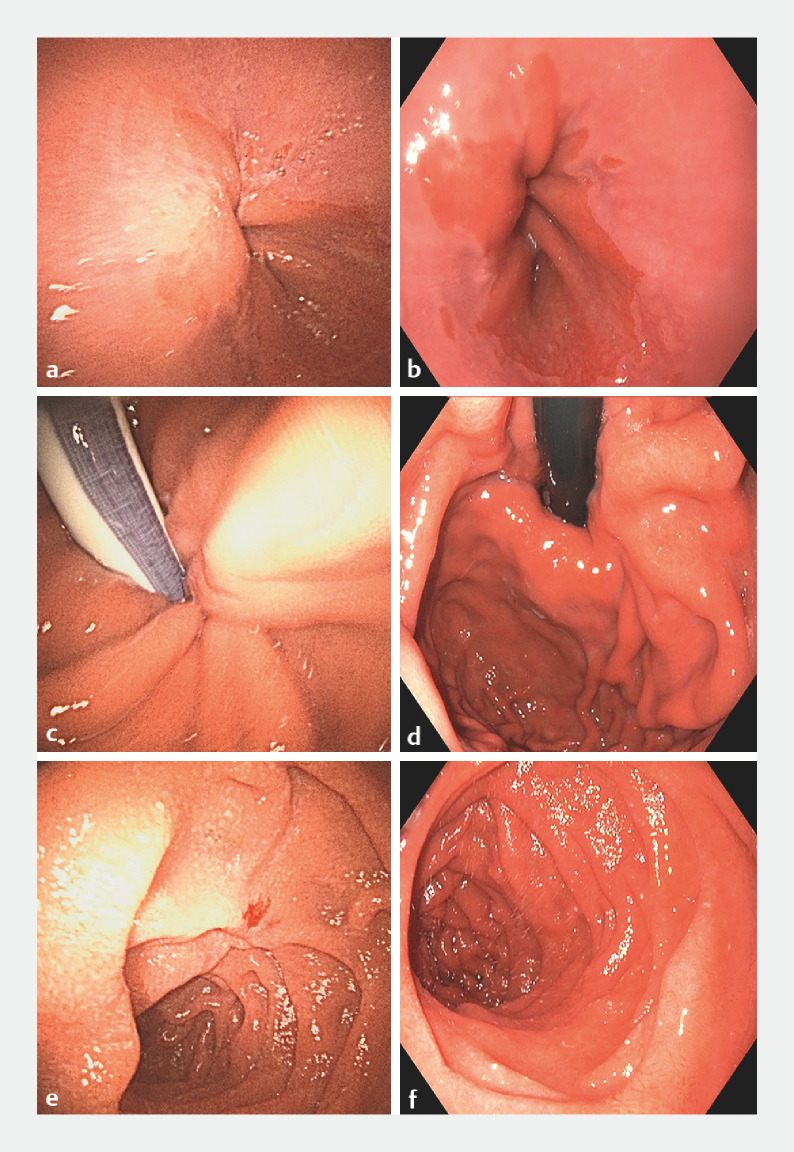
Comparative images from a single patient, taken during two consecutive esophagogastroduodenoscopies performed for the same indication: analysis of lymphadenopathies with progressive clinical deterioration. The images on the left ( **a** , **c** , **e** ) were obtained with an Ambu aScope Gastro single-use gastroscope, whereas the images on the right ( **b** , **d** , **f** ) were acquired using an Olympus 180 series gastroscope.

In the Erasmus MC, 18 of 20 performance characteristics were rated as “comparable” or “better” in at least 85% of EGDs. The weight of the single-use gastroscopes was rated as better than the reusable scopes in 50% of cases. Duodenal intubation proved more challenging in 35% of EGDs, whereas picture quality was deemed inferior in 47% of cases. Fig. 2 presents a comparative display of images captured using an Ambu aScope Gastro and an Olympus 180 series gastroscope. At the OUS, 11 of 20 performance characteristics were rated as “comparable” or “better” in 85% of EGDs. Notably, duodenal intubation was rated as better than reusable gastroscopes in 33% of cases, and weight was scored as better than reusable in all instances. Furthermore, fit to hand, maneuverability, and distal tip deflection were considered as better than reusable scopes in over 50% of cases. However, image size was rated as inferior in 100% of EGDs. Also, other characteristics related to image quality, such as camera focus, lighting, color reproduction, and overall picture and video quality, were rated as inferior compared with reusable scopes in at least 35% of procedures. In the general remarks left by the endoscopists from the Erasmus MC, common points of feedback were the absence of a freeze button (16 cases), lens cleaning problems (8 cases), and extensive loop formation in the stomach (8 cases). The endoscopists from OUS reported that the screen size was too small (6 cases). Overall satisfaction was rated as “comparable” in 87% of the EGDs at Erasmus MC and 96% at OUS. Importantly, there were no device failures or adverse events.

## Discussion

In this series of 60 patients, upper gastrointestinal endoscopy with a single-use gastroscope could be accomplished in a satisfactory percentage of almost 97% of cases. There was no limitation in the execution of standard diagnostic and therapeutic procedures while using a single-use endoscope except for two cases with a non-traversable esophageal stricture. It is important to note that these two crossovers were necessitated by the single-use gastroscope diameter, which matches that of reusable gastroscopes. Therefore, these two cases do not indicate a performance failure. Overall, handling of the single-use gastroscopes was rated at least comparable to reusable gastroscopes. Performance characteristics related to video and picture quality, however, were rated lower.

This study shows that single-use gastroscopes could be used for a myriad of standard indications, offering a proper alternative to reusable gastroscopes. This is supported by a recent publication, which demonstrated successful treatment of six cases of upper gastrointestinal bleeding using the Ambu single-use gastroscope 19 . The use of single-use endoscopes for patient treatment is a topic of ongoing debate. While they offer significant advantages, such as eliminating the need for reprocessing and reducing gastroscope-associated infections, concerns have emerged regarding their environmental impact. In an era in which climate change necessitates environmental responsibility, it should also guide decisions in health care in general and, for this subject in particular, the Endoscopy Department 14 . Furthermore, cost implications of implementing single-use gastroscopes require further exploration. The current study solely focused on performance of single-use gastroscopes and did not include environmental or cost aspects of implementing these endoscopes in daily practice.

Single-use gastroscopes have certain areas for improvement. First, their image quality was impacted by lens cleaning difficulties, and the absence of a freeze button impeded documentation. Furthermore, lack of a narrow band imaging function impairs lesion characterization and assessment of intestinal metaplasia 20 . However, the inherent benefit of single-use endoscopes lies in their adaptability, allowing fast incorporation of design and functional improvements in newer versions. This adaptability enables early availability of new functions in clinical practice and for research purposes. Hospitals and clinics are not bound by the long-term investment in reusable endoscopes. Instead, they can promptly adopt newer versions of single-use gastroscopes when they become available.

There were several differences between the patient populations and procedure characteristics at Erasmus MC and OUS. In OUS, patients were typically younger, predominantly male, and more frequently under general anesthesia, contributing to their increased comfort during the procedure. Regarding procedure characteristics, a larger number of endoscopists performed the EGDs, procedure times were longer, and the primary indication was often therapeutic. Erasmus MC also employed multiple screens during EGDs, as opposed to just the aBox 2 screen. These distinctions can largely be attributed to the distinct settings: ICU versus the standard Endoscopy Department. For instance, epidemiological studies have indicated a higher ICU admission rate for men 21 . In addition, Erasmus MC required informed consent for participation, potentially introducing inclusion bias. These numerous variations may have influenced evaluation of single-use gastroscopes. Lower weight, for instance, potentially proved more advantageous during longer procedures and use of multiple (larger) screens might explain the difference in image quality rating between the study centers.

While the results of this case series are promising, this study has some limitations. No control group was available and the performance rating was predominantly based on subjective factors, which was underscored by the difference in ratings between the study centers. Although we included patients from different departments and with a broad range of indications, we cannot claim generalizability to the total population of patients undergoing EGD. In addition, all EGDs were conducted by highly experienced endoscopists. Consequently, our findings cannot be readily generalized to novice or trainee endoscopists. Series from other groups are needed to confirm the effectiveness and safety of single-use gastroscopes compared with reusable gastroscopes in clinical practice.

## Conclusions

This series shows that single-use gastroscopes can be used successfully for a broad range of indications. Potential benefits are prevention of endoscope-associated infections, absence of reprocessing time, and improved workflow when performing endoscopy in remote locations. These benefits must be weighed against costs and environmental impact. To make informed decisions regarding implementation of single-use gastroscopes in endoscopy practice, a better understanding of their environmental impact in comparison with reusable endoscopy is needed.

## Notes

Correction **Correction: Performance of a single-use gastroscope for esophagogastroduodenoscopy: Prospective evaluation** Koen van der Ploeg, Pieter J.F. de Jonge, Wim J. Lammers et al. Endoscopy International Open 2024; 12: E428–E434. DOI: 10.1055/a-2271-2303 In the above-mentioned article Figure 1 was corrected. This was corrected in the online version on 13.09.2024.
