# Correction: Performance of a single-use gastroscope for esophagogastroduodenoscopy: Prospective evaluation

**DOI:** 10.1055/a-2412-6842

**Published:** 2024-09-13

**Authors:** Koen van der Ploeg, Pieter J.F. de Jonge, Wim J. Lammers, Arjun Dave Koch, Margreet C. Vos, Vemund Paulsen, Lars Aabakken, Marco Bruno

**Affiliations:** 1 6993 Department of Gastroenterology and Hepatology, Erasmus MC, Rotterdam, Netherlands; 2 6993 Department of Medical Microbiology and Infectious Diseases, Erasmus MC, Rotterdam, Netherlands; 3 72481 Department of Gastroenterology and Hepatology, Erasmus MC Cancer Centre, Rotterdam, Netherlands; 4 155272 Department of Clinical Medicine, Oslo University Hospital, Oslo, Norway; 5 6305 Institute of Clinical Medicine, University of Oslo, Oslo, Norway; 6 155272 Department of Transplantation Medicine, Oslo University Hospital, Oslo, Norway



In the above-mentioned article Figure 1 was corrected. This was corrected in the online version on 13.09.2024.

